# Heme Oxygenase-1 mRNA Expression in Egyptian Patients With Chronic Liver Disease

**DOI:** 10.5812/hepatmon.846

**Published:** 2012-04-30

**Authors:** Sahar Saad El-Din Bessa, Ehab Mostafa Mohamed Ali, Abeer El-Sayed Abd El-Wahab, Sherif Abd El-Monem Nor El-Din

**Affiliations:** 1 Department of Internal Medicine, Faculty of Medicine, Tanta University, Tanta, Egypt; 2 Departments of Chemistry, Division of Biochemistry, Faculty of Science, Tanta University, Tanta, Egypt; 3 Department of Medical Biotechnology, Genetic Engineering and Biotechnology Research Institute, The Scientific Research´s City and Technology Applications, Alexandria, Egypt

**Keywords:** Gene Expression, Heme Oxygenase-1, Hepatitis C Virus, Liver Cirrhosis, Non-alcoholic Fatty Liver Disease, Oxidative Stress

## Abstract

**Background:**

Chronic liver disease (CLD) is a global medical problem. This disease is associated with increased hepatic oxidative stress. One of the antioxidant enzymes that protect cells against this stress is heme oxygenase-1 (HO-1).

**Objectives:**

This study aimed to investigate the mRNA expression of HO-1 in Egyptian patients with CLD and its relation to oxidative stress biomarkers.

**Patients and Methods:**

Levels of serum ferritin, carboxyhemoglobin, malondialdehyde (MDA), and erythrocyte-reduced glutathione (GSH) were measured, and HO-1 mRNA expression was detected in 45 CLD patients (15 with nonalcoholic steatohepatitis [NASH], 15 with chronic hepatitis C, and 15 with liver cirrhosis) and 15 healthy controls.

**Results:**

HO-1 mRNA expression was increased in patients with NASH, chronic hepatitis C, and liver cirrhosis compared to controls. The expression in cirrhotic patients was significantly higher than that in patients with NASH and chronic hepatitis C. Compared to controls, patients with NASH, chronic hepatitis C, and liver cirrhosis had higher levels of ferritin, carboxyhemoglobin, and MDA and lower levels of GSH. HO-1 mRNA expression was positively correlated with levels of carboxyhemoglobin, serum ferritin, and serum MDA and negatively correlated with levels of erythrocyte GSH in CLD patients.

**Conclusions:**

HO-1 mRNA expression was significantly increased in CLD patients, and the increase reflected the severity of the disease. The significant relationship between the increased HO-1 expression and oxidative stress biomarkers in patients with CLD suggests that HO-1 may play an important role in protecting the liver from oxidative stress-dependent damage. Therefore, induction of HO-1 could be a novel therapeutic option for CLD.

## 1. Background

Chronic liver disease (CLD) is an important cause of morbidity and mortality and represents a major health problem worldwide. Liver cirrhosis is the final common pathway of many cases of CLD; however, the exact point at which a disease process produces cirrhosis is difficult to define [1]. Hepatitis C virus (HCV) infection causes liver disease characterized by inflammation, cell damage, and progressive liver fibrosis leading to cirrhosis [2]. Non-alcoholic steatohepatitis (NASH) is a clinical-pathological condition characterized by a necroinflammatory disorder with fatty infiltration of hepatocytes and may progress to fibrosis or cirrhosis, with a fatal outcome [3]. Although the precise molecular mechanisms by which HCV causes liver injury are not fully understood, recent evidence demonstrates that HCV causes oxidative stress in human liver cells, due to the generation of reactive oxygen species (ROS) [4][5][6]. Moreover, oxidative stress and lipid peroxidation play an important role in the pathogenesis of NASH, as their end products can induce hepatocellular injury and fibrogenesis [7][8][9]. The liver is the major organ that detoxifies excess heme molecules by the action of the heme oxygenase (HO) enzyme that catalyzes the initial and rate-limiting reaction in heme catabolism and cleaves pro-oxidant heme to form iron, carbon monoxide (CO), and biliverdin, which is subsequently converted to bilirubin by biliverdin reductase [10]. The liver contains 2 HO isozymes for physiologic degradation of the heme; HO-1 is inducible various stimuli, including cytokines, heavy metals, and reactive oxygen species, while HO-2 is constitutively produced. The antioxidant property of HO is derived from the elimination of pro-oxidant heme and from the biological activities of its reaction products: biliverdin, bilirubin, and iron [11]. Overproduction of biliverdin and bilirubin serves as an antioxidative defense mechanism [12]. Free iron inhibits the de novo synthesis of heme, binds to iron regulatory protein, and stimulates the biosynthesis of ferritin, which exerts an additional antioxidant effect. Another important role of the HO reaction in regulating liver function is to generate CO, which has anti-inflammatory effects and acts as an endogenous regulator to maintain microvascular blood flow [13]. CO regulates the hepatic vascular tone in a variety of experimental models of conditions such as ischemia-reperfusion, endotoxemia, and heme overloading [14][15]. Recently, much attention has been paid to physiologic and pathophysiologic roles of the HO enzyme in organ homeostasis [16]. HO-1 has potent cytoprotective effects; it is an anti-oxidant defense enzyme that converts potentially toxic heme into anti-oxidants [17] and is a stress-responsive protein that is essential for higher eukaryotes to cope with cellular stress and to regulate cellular iron metabolism [18][19]. Considering these findings, we investigated the relation between HO-1 mRNA expression in Egyptian patients with CLD and oxidative stress biomarkers.

## 2. Objectives

This study investigated the mRNA expression of HO-1 in Egyptian patients with CLD and the relation of HO-1 expression with oxidative stress biomarkers.

## 3. Patients and Methods

### 3.1. Patients

We randomly selected 45 Egyptian patients with CLD from those admitted to the Internal Medicine Department in the Tanta University Hospital between April 2010 and March 2011. The cohort consisted of 15 patients with NASH (6 men and 9 women) with a mean age of 53 ± 8.9 years, 15 patients with chronic hepatitis C (6 men and 9 women) with a mean age of 52 ± 8.2 years, and 15 patients with liver cirrhosis (8 men and 7 women) with a mean age of 54.5 ± 7.6 years. The study protocol was approved by the General Ethics Council of the Tanta University Hospital, Tanta University, Tanta, Egypt, and all patients gave their informed consent to participate in the study. The diagnosis of NASH was established on the basis of the following clinical and histopathological features: (i) abnormal liver biochemistry for more than 3 months; (ii) liver biopsy showing steatosis (10%) in the presence of lobular and/or portal inflammation, with or without Mallory bodies or fibrosis; and (iii) the exclusion of other liver diseases. Diagnosis of chronic hepatitis C was established by serological detection of anti-HCV antibodies and HCV-RNA, and serum transaminase levels greater than 3 times the upper limit of normal for at least 6 months, and confirmed by liver biopsy. The diagnosis of liver cirrhosis was established on the basis of clinical, biochemical, histological, and ultrasound findings. The etiology of cirrhosis was HCV-related in 8 patients, NASH in 4, and cryptogenic in 3 patients. In addition, all cirrhotic patients had schistosomal periportal hepatic fibrosis. Severity of the liver disease was scored according to Child-Pugh classification [20]. Other causes of chronic liver disease, including drugs, alcohol, chronic hepatitis B virus (HBV) infection, autoimmune liver disease, hereditary hemochromatosis, Wilson disease, and α-1 antitrypsin deficiency, were excluded by appropriate serological studies and findings for liver biopsy samples. Hepatocellular carcinoma was ruled out by abdominal ultrasound, spiral abdominal computed tomography, and alpha fetoprotein determination. Patients did not receive antioxidant vitamin or selenium supplementation within the 2 months preceding their inclusion in the study.

### 3.2. Controls

Fifteen healthy age- and sex-matched volunteers (9 men and 6 women) with a mean age of 50 ± 3.9 years were recruited for participation as controls. They were selected from medical and paramedical staff and from blood donors in the Tanta University Hospital. They gave informed consent to participate in the study. They were seronegative for viral hepatitis markers, and for none of them, liver function tests yielded abnormal results.

### 3.3 Blood Collection and Biochemical Analysis

Fasting blood samples were obtained aseptically from patients and controls and divided into 3 parts; one part was collected in a vacutainer plain tube without anticoagulant, incubated at 37 ºC for half an hour, and centrifuged at 3,000 ×g for 10 min. This serum sample was used for in determination of liver function tests, including clinical chemistry, alpha fetoprotein estimation by radioimmunoassay, anti-HCV antibodies detection using commercially available enzyme-linked immunoassay along with serum HCV RNA determination by reverse transcription-polymerase chain reaction (RT-PCR), HBV surface antigen and HBV core antibodies detection by commercial assays, determination of ferritin levels using a kit supplied from Biosystems [21], determination of lipid peroxidation end-product and malondialdehyde (MDA) levels by the thiobarbituric acid method [22]. The second fraction was drawn into ethylenediaminetetraacetic acid (EDTA) tubes for estimation of the percent saturation of hemoglobin by carbon monoxide, i.e., carboxyhemoglobin (COHb) concentration, as an indicator of HO-1 activity, using spectrophotometry as previously described [23]. The erythrocyte pellet was washed 3 times with cold isotonic saline and then diluted with saline to the original blood volume. Hemoglobin concentration was determined using a kit supplied by BioMeriux, France. The erythrocyte-reduced GSH level was measured as previously described [24] and expressed as mmol/g Hb. The third part was collected in a heparin sterile vacutainer for determination of HO-1 mRNA expression by RT-PCR. Total RNA was extracted from peripheral blood mononuclear cells (PBMCs) using a total RNA isolation system from Promega Corporation, Madison, USA [25]. RT-PCR reaction for HO mRNA was performed using QIAGEN’s 1-step RT-PCR kit (Hilden, Germany), according to the manufacturer’s instructions. The final concentrations of the reaction components (50 µL) were 1× QIAGEN buffer, 400 µM of each dNTP, 0.6 µM of both sense and antisense HO primers, 2 µL of RT-PCR enzyme mix, 5 units of RNase inhibitor, and 2 µg of total RNA. Reverse transcription was achieved by heating the reaction components at 50 ºC for 30 min. The initial PCR activation step was performed by heating at 95 ºC for 15 min. The amplification reaction was carried out by thermal cycler (model 9600, Perkin-Elmer) for 35 cycles of denaturation at 94 ºC for 1 min, annealing at 55 ºC for 1 min, and extension at 72 ºC for 1 min, followed by a final extension step of 72 ºC for 10 min. The HO complementary DNA (cDNA) was amplified using the following primers: sense, 5′-CAGGCAGAGAATGCTGAGTTC-3′ and antisense, 5′-GATGTTGAGCAGGAACGCAGT-3′ [26]. ß-actin was used as a housekeeping gene with the following primers: sense, 5›-TTCTTTGCAGCTCCTTCGTTGCCG-3› and antisense, 5›-TGGATGGCTACGTACATGGCTGGG-3›. The amplified product for HO was separated on 2% agarose gel and visualized by ethidium bromide staining under ultraviolet light. Primers for HO were synthesized by Metabion International AG (Martinsried, Germany). The RT-PCR reactions were performed with a Hybaid thermal cycler (Thermo Electron corporation [formerly Hybaid], Waltham, MA, USA). The molecular weight was determined using a DNA ladder purchased from Promega (Madison, WI, USA). The computer program “Quantity one” (version 4.6.3, Bio-Rad, USA) was used to analyze the intensities of the PCR bands.

### 3.4. Statistical Analysis

Results were expressed as mean ± standard deviation (SD). Comparisons between groups were made using Student´s t-test for continuous variables. The correlation between 2 parameters was determined by Pearson’s correlation coefficient (r). A P value of less than 0.05 was considered statistically significant.

## 4. Results

**Figure 1:**
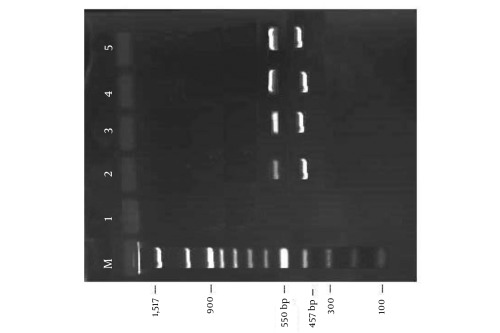
Representative Agarose Gel Electrophoresis for Amplified PCR Product of Heme Oxygenase-1 Gene at 550 bp and ß-actin at 457 bp as a Housekeeping Gene. Lane M, DNA molecular marker; Lane 1, blank; Lane 2, Control; Lane 3, nonalcoholic steatohepatitis; Lane 4, chronic hepatitis C; Lane 5, liver cirrhosis (bp = base pair)

The main clinical and biochemical characteristics of patients with CLD are presented in Table 1. HO-1 mRNA expression was determined by RT-PCR, as shown in Figure 1. The densities of the bands are expressed in arbitrary units.

**Table 1:** Clinical and Biochemical Characteristics of Patients With Chronic Liver Disease and Controls

**Characteristic**	**Controls (n = 15)**	**NASH n (n = 15)**	**HCV n (n = 15)**	**Liver Cirrhosis (n = 15)**
Age, y	50 ± 3.9	53 ± 8.9	52 ± 8.2	54.5 ± 7.6
Gender				
Male	9	6	6	8
Female	6	9	9	7
Child-Pugh				
Class A	-	11	6	1
Class B	-	4	9	3
Class C	-	0	0	11
ALT n, U/L, mean ± SD	23.87 ± 5.05	50.6 ± 20.3^a^	60.4 ± 23.53 d	62.4 ± 25.6 i
AST n, U/L, mean ± SD	22.77 ± 4.06	48.53 ± 20.34^a^	55 ± 21 d	80.67 ± 30.72 i,k,m
Serum bilirubin, mg/dL, mean ± SD	0.58 ± 0.22	0.8 ± 0.21^c^	1.12 ± 0.52^e^,^h^	1.78 ± 1.07^i^,^k^,^m^
Serum albumin, g/L, mean ± SD	43.5 ± 2.2	42.2 ± 2.8	41.7 ± 3.5	29.3 ± 3.4^i^,^j^,^l^
Prothrombin activity, %, mean ± SD	95 ± 3.31	93 ± 3.9	92 ± 4.8	72.2 ± 6.5 i,j
Serum ferritin , ng/mL, mean ± SD	92.13 ± 59.46	1.25 ± 0.3 b	309.8 ± 92.19 d,g	845.27 ± 129.97 i,j,l
Carboxyhemoglobin , %, mean ± SD	0.96 ± 0.13	1.25 ± 0.3 b	1.84 ± 0.43 d	2.33 ± 0.53 i,j,m
MDA n, µmol/L, mean ± SD	1.12 ± 0.19	1.51 ± 0.24^a^	2.09 ± 0.4 d,f	2.49 ± 0.6 i,j,m
GSH n, µmol/g Hb, mean ± SD	4.55 ± 0.24	4.06 ± 0.11^a^	3.79 ± 0.13 d,l^f^	3.39 ± 0.21^i^,^j^,^l^
HO-1 n mRNA expression (arbitrary units), mean ± SD	110.4 ± 26.63	130 ± 24.06 c	168.47 ± 20.16 d	207.73 ± 25.54 i,j,l

^a^ P < 0.001

^b^ P < 0.01

^c^ P < 0.05 NASH vs. controls

^d^ P < 0.001

^e^ P < 0.01 HCV vs. controls

^f^ P < 0.001

^g^ P < 0.01

^h^ P < 0.05 HCV vs. NASH

^i^ P < 0.001 liver cirrhosis vs. controls

^j^ P < 0.001

^k^ P < 0.01 liver cirrhosis vs. NASH

^l^ P < 0.001

^m^ P < 0.05 liver cirrhosis vs. HCV

^n^ Abbreviations: ALT, alanine transaminase; AST, aspartate transaminase; GSH, reduced glutathione; HCV, hepatitis c virus; HO-1, heme oxygenase-1; MDA, malondialdehyde; NASH, non-alcoholic steatohepatitis

**Figure 2:**
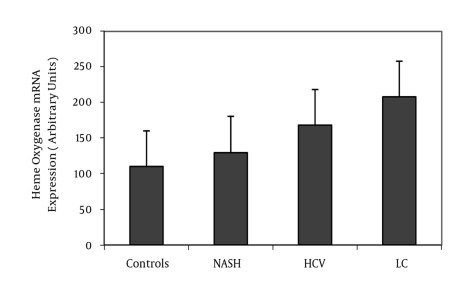
Mean + S. D. of Heme Oxygenase-1 mRNA Expression in Patients With Chronic Liver Disease and Controls

**Figure 3:**
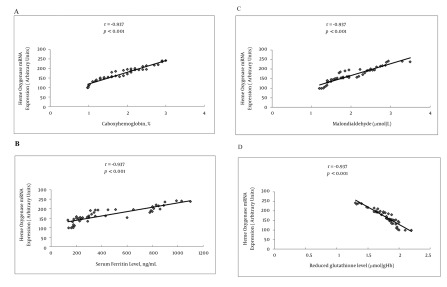
Correlation Between Heme Oxygenase-1 mRNA Expression and Carboxyhemoglobin Concentration (A), Serum Ferritin Level (B), Serum Malondialdehyde Level (C) and Reduced Glutathione Level (D) in Patients With Chronic Liver Disease

HO-1 mRNA expression in patients with NASH, chronic hepatitis C, and liver cirrhosis was more than that in controls (P < 0.05, P < 0.001, and P < 0.001, respectively). Further, compared to patients with NASH and chronic hepatitis C, patients with cirrhosis had significantly higher HO expression (P < 0.001, for both). HO-1 mRNA expression in chronic hepatitis C patients was significantly more than that in patients with NASH (P < 0.001), as shown in Table 1 and Figure 2. Serum ferritin, carboxyhemoglobin, and serum MDA levels in patients with NASH, chronic hepatitis C, and liver cirrhosis were significantly higher than the levels in healthy controls. These levels were elevated in cirrhotic patients compared to NASH and chronic hepatitis C patients. Moreover, the levels in patients with chronic hepatitis C were higher than those in patients with NASH (P < 0.01, P < 0.001, and P < 0.001, respectively). On the other hand, erythrocyte GSH levels were decreased in patients with NASH, chronic hepatitis C, and liver cirrhosis compared to controls (P < 0.001, for all) and were significantly lower in cirrhotic patients than in NASH and chronic hepatitis C patients (P < 0.001, for both). Moreover, the erythrocyte GSH level in patients with chronic hepatitis C was significantly lower than those in patients with NASH (P < 0.001) as shown in Table 1. Table 2 shows that the HO-1 mRNA expression in Child-Pugh class C was more than that in Child-Pugh class A and B (P < 0.001 and P < 0.05, respectively). Moreover, serum ferritin, carboxyhemoglobin, and serum MDA levels in Child-Pugh class C were significantly higher than those in class A and B. In contrast, erythrocyte GSH level in Child-Pugh class C was lesser than that in class A and B (P < 0.001, for both). In the present study, HO-1 mRNA expression was positively correlated with levels of carboxyhemoglobin (r = 0.963, P < 0.001), serum ferritin (r = 0.868, P < 0.001), and serum MDA (r = 0.978, P < 0.001) and negatively correlated with erythrocyte GSH level (r = -0.937, P < 0.001) in CLD patients, as shown in Figure 3.

**Table 2:** Ferritin Level, Carboxyhemoglobin Concentration, Malondialdehyde (MDA) Level, Reduced Glutathione (GSH) Level and Heme Oxygenase-1 (HO-1) mRNA Expression Among Patients With Chronic Liver Disease According to Child-Pugh Classification

	**Class A (n=45**)****	**Class B (n=45)**	**Class C (n=45)**
Patients, No.(%)	18/45 (40)	16/45 (35.6)	11/45 (24.4)
Serum ferritin, ng/mL, mean ± SD	280.83±134.44	736.56±162.96 a	928.33±129.54 c,e
Carboxyhemoglobin, %, mean ± SD	1.54±0.47	2.23±0.52 a	2.63±0.47^c^,^f^
MDA, µmol/L, mean ± SD	1.79±0.43	2.37±0.5^b^	2.85±0.6 c,f
GSH, µmol/g Hb, mean ± SD	3.94±0.13	3.47±0.2 a	3.01±0.21 c,d
HO-1 mRNA expression, arbitrary units, mean ± SD	149.87±29.76	197.78±27.77^a^	219.5±23.05^c^,^f^

^a^ P < 0.001

^b^ P < 0.01 class B vs. class A

^c^ p < 0.001 class C vs. Class A

^d^ p < 0.001

^e^ p < 0.01

^f^ P < 0.05 class C vs. class B

## 5. Discussion

HO-1 is an enzyme that catalyzes the rate-limiting step in heme degradation to result in the formation of iron, carbon monoxide, and biliverdin, which is subsequently converted to bilirubin by biliverdin reductase. The biological effects exerted by the products of this enzymatic reaction have gained much attention. The anti-oxidant, anti-inflammatory, and cytoprotective functions associated with HO-1 are attributable to 1 or more of its degradation products. Induction of HO-1 occurs as an adaptive and beneficial response to various injurious stimuli such as oxidative stress, and this inducible nature of HO-1 signifies its importance in several pathophysiological states such as liver diseases. Thus, HO-1 has emerged as a key target molecule with therapeutic implications [27]. This study investigated the mRNA expression of HO-1 in Egyptian patients with CLD and its relation to oxidative stress biomarkers, and we showed that HO-1 mRNA expression was increased in patients with NASH, chronic hepatitis C, and liver cirrhosis compared to controls. HO-1 expression in cirrhotic patients was significantly higher than that in patients with NASH and chronic hepatitis C. The HO-1 mRNA expression was increased in Child-Pugh class C compared to Child-Pugh class A and B. Furthermore, HO-1 mRNA expression in CLD patients was positively correlated with levels of carboxyhemoglobin, serum ferritin, and serum MDA and negatively correlated with level of erythrocyte GSH. These results are in agreement with the findings of Malaguarnera et al. [28], who showed that HO-1 expression was significantly increased in NASH patients, and the increase reflected the severity of the disease. They observed a significant correlation between the increased levels of HO-1 and ferritin and between the increased levels of HO-1 and lipid peroxidation. Moreover, NASH patients with lower levels of GSH exhibited higher expression of HO-1. Thus, the induction of HO-1 is an adaptive response against oxidative damage elicited by lipid peroxidation, and this may be critical in the progression of the disease. This was supported by Nan et al. [29], who reported that HO-1 plays an important role in NASH. Regarding alcoholic steatohepatitis, Yao et al. [30] have shown that the induction of HO-1 by Ginkgo biloba is associated with a decrease in liver damage caused by ethanol feeding for 90 d in rats. This is probably due to the enhanced anti-oxidative capacity against ethanol-induced oxidative stress and maintenance of cellular redox balance [31][32]. Conflicting data are available on HO-1 in hepatitis C. Ghaziani et al. [33] demonstrated that HCV-expressing human hepatoma cells have increased levels of HO-1 and decreased Bach 1 expression. Abdalla et al. [34], on the contrary, observed a decrease in HO-1 and HO-1 mRNA in liver biopsies from HCV-infected patients. The expression of HO-1 was also reduced in cell lines that stably express the HCV core protein, which suggests that core gene products are capable of regulating HO-1 expression. Clearly, further studies are necessary to reconcile these conflicting results. Concerning the effects of HO-1 induction on hepatitis C, Shan et al. [35] have shown a decrease in HCV replication, an effect similar to that described by Protzer et al. [36], in HBV infection. Moreover, Zhu et al. [37] showed that targeted overexpression of HO-1 led to a significant inhibition of HCV replication without affecting other parameters of cell viability in human hepatoma cells. These studies have recently been extended by others, who have shown that the HO-1 product biliverdin interfered with HCV replication via direct modulation of the antiviral interferon-α response in 2 human hepatoma replicon cell lines [38]. Thus, HO-1 might serve as a specific therapeutic target for the treatment of chronic HCV infection, although the results are somewhat contradictory. With respect to liver cirrhosis, Wei et al. [39] have shown that HO-1 mRNA and protein expression is increased in hepatocytes and some Kupffer cells in the early phase of the disease, while HO-2 expression in these cells is unchanged. In cirrhotic livers, mainly those with biliary cirrhosis, both HO-1 and HO-2 are increased, as shown by Goh et al. [40]. HO-1 was localized mainly in Kupffer cells, while HO-2 was localized in the cytoplasm of the hepatocytes. Similar results were obtained by Makino et al. [41] in patients with post-hepatic cirrhosis. They showed that HO-1 expression increased in the liver, being mainly distributed in Kupffer cells and hepatocytes. Moreover, a study on cirrhotic patients undergoing liver transplantation has shown that HO-1 is upregulated through heme-independent stimuli, according to the development of portal hypertension, and that induced HO-1 plays a pathophysiological role in portal hypertension through CO production [42]. In the current study, carboxyhemoglobin concentration in patients with NASH, chronic hepatitis C, and liver cirrhosis was significantly higher than that in healthy controls. The concentration was elevated in cirrhotic patients compared to NASH and chronic hepatitis C patients. These results are consistent with the findings of previous studies [43][44]. In cirrhotic patients with spontaneous bacterial peritonitis, CO production, evaluated as the CO concentration in the exhaled air and blood CO-Hb level, is further increased and may participate in circulatory alterations [23]. In our study, levels of serum ferritin in patients with NASH, chronic hepatitis C, and liver cirrhosis were significantly higher than those in healthy controls. High ferritin levels have previously been reported in NASH [28] and chronic HCV-related hepatitis [45][46], and our study clearly confirms this finding. An imbalance in the pro-oxidant/antioxidant equilibrium in favor of pro-oxidants constitutes the oxidative stress phenomenon, a condition that may induce a number of pathophysiological events in the liver [9]. In this study, serum MDA level was significantly higher and erythrocyte GSH level was significantly lower in patients with NASH than in healthy controls. Similar findings were reported by previous studies [7][28][47]. Meanwhile, we also observed that compared to healthy controls, patients with chronic hepatitis C had higher serum MDA and lower erythrocyte GSH levels. These results are in accordance with the findings of other studies [4][6][48]. The present study provided evidence for the protective role of HO-1 in the body’s response to oxidative stress. Due to its beneficial effects, the targeted induction of HO-1 is considered to have major therapeutic potential for the treatment of inflammatory liver diseases to prevent progression of early stages of liver injury [49]. In this context, it is interesting to note that induction of HO-1 expression by natural compounds contributes to protection against liver damage in various experimental models [50]. Current knowledge on the applications of HO-1 as a therapeutic target still seems precocious and critical questions remain to be answered before clinical interventions become available. Because induction of HO-1 expression may hold therapeutic promises, studies designed to identify clinically relevant methods of delivering such treatment, such as gene therapy to increase the induction of HO-1 or methods to deliver individual by-products of heme degradation by HO (e.g., CO and bilirubin), would be warranted. From this study, we conclude that HO-1 mRNA expression is significantly increased in CLD patients, and the increase reflects the severity of the disease. The significant relationship between the increased HO-1 expression and oxidative stress biomarkers in patients with CLD suggest that HO-1 may play an important role in protecting the liver from oxidative stress-induced damage. Therefore, the induction of HO-1 might be a novel therapeutic option for CLD. However, large-scale clinical studies are needed to better clarify the exact role of the heme-HO system in CLD and the potential clinical applications of inducing the HO-1 system.
